# Correlation between mechanism of oxidized-low density lipoprotein-induced macrophage apoptosis and inhibition of target gene platelet derived growth factor receptor-β expression by microRNA-9

**DOI:** 10.1080/21655979.2021.2006864

**Published:** 2021-12-11

**Authors:** Xunan Guo, Yan Chai, Yuqing Zhao, Dongying Wang, Peng Ding, Yunfei Bian

**Affiliations:** a Department of Cardiology, The Second Hospital of Shanxi Medical University, Taiyuan, China; b Department of Cardiology, Xinzhou People’s Hospital, Xinzhou, China; c Digestive System Department, The Second Hospital of Shanxi Medical University, Taiyuan, China

**Keywords:** Oxidized-low density lipoprotein, macrophages, microRNA-9, platelet derived growth factor receptor-β

## Abstract

This study was to explore the effects of oxidized-low density lipoprotein (ox-LDL) on the proliferation and apoptosis of macrophages, and the role of miRNA-9 in the targeted regulation of platelet-derived growth factor receptor-β (PDGFR-β) expression. Macrophage RAW264.7 cells were cultured and foamed with 100 mg/L ox-LDL to detect the cell proliferation and apoptosis and target protein expression levels. Subsequently, the miRNA-9 mimics and inhibitors were transfected to detect the expression level of PDGFR-β. The dual-luciferase reporter gene was predicted and applied to detect the target-binding effect of miRNA-9 and PDGFR-β in the cells. The results showed that ox-LDL could induce the foaming of macrophages RAW264.7, inhibit the cell proliferation, and promote the cell apoptosis. After ox-LDL induction, expression of Caspase-3 in macrophages RAW264.7 was up-regulated, and that of glucose regulated protein 78 was down-regulated. The transfection of miRNA-9 mimics could greatly inhibit the expression of PDGFR-β mRNA and proteins in the cells. In addition, the results of the dual-luciferase reporter gene showed that the ratio of luciferase activity was significantly reduced after the miRNA-9 mimic and the wild-type PDGFR-β plasmid were co-transfected. In summary, ox-LDL could induce foaming of macrophages and promote cell apoptosis, and miRNA-9 could target and bind to the 3ʹUTR region of PDGFR-β, thereby inhibiting the gene expression.

## 1. Introduction

Atherosclerosis (AS) [1] is a disease mainly characterized by vascular endothelial injury, lipid infiltration, and chronic inflammation. Macrophages affect the progression of AS lesions through their effects on lipid content, inflammatory response, degradation of fibrous components, and formation of new blood vessels in plaques [2]. Studies found that macrophage apoptosis occurs at all stages of AS. In the early stage of the disease, macrophage apoptosis can reduce the number of plaque cells and the degradation of extracellular collagen due to the normal clearance ability of apoptotic cells, thus inhibiting the progression of AS [3]. However, macrophage apoptosis increases and the ability to remove apoptotic cells decreases with the development of the disease, which may cause inflammatory reaction and increase lipid deposition. In particular, the release of protease after macrophage apoptosis can damage the fibrous cap and affect the stability of plaque. Therefore, intervention in the apoptosis of macrophages is of great significance to prevent the progress of AS [4]. Studies showed that oxidized-low density lipoprotein (ox-LDL) can inhibit macrophages, but the specific mechanism is not clear. Platelet derived growth factor (PDGF) family plays a key role in normal embryonic development, cell growth and differentiation, and response to collective tissue damage. Many pathological processes are related to the activity of PDGF and its receptor. Platelet derived growth factor receptor (PDGFR), an important member of casein kinase family, can promote cell proliferation, differentiation, invasion, and migration. PDGF is the important link in AS. It is to promote from arterial smooth muscle cells migrate to the inner membranes and appreciation of the important factors. In addition, it can also be induced from the transcription matrix metalloproteinases (MMPs) synthesis, and accelerate the degradation of extracellular matrix, eventually leading to the formation of atherosclerosis. It is a widely expressed non-coding RNA with a length of 17–24 nucleotides, which plays the role of gene silencing by inhibiting gene translation and/or promoting the degradation of its miRNA [5]. MicroRNAs are ubiquitous in human, nematode, and plant cells. Regulation of microRNA genes, target genes finding, and their mechanism of action revealing are very important. The miRNA-9 can regulate tumor apoptosis by silencing kinds of apoptosis-related genes or regulating key molecules of apoptosis-related signaling pathways [6].

This study was to explore the mechanism of ox-LDL inducing macrophages and the role of miRNA. In addition, it explored how ox-LDL induced apoptosis of macrophages and the correlation between miRNA-9 inhibition of target gene PDGFR-β expression. RAW264.7 cells were taken as the research object, and the degree of apoptosis and the activity of caspase-3 were detected by culture and treatment. The miRNA-9 mimics and inhibitors were transfected to detect their effects on the expression of PDGFR-β in macrophages, and predict and detect the targeting effects of miRNA-9 on PDGFR-β. This study aimed to provide reference for the mechanism of macrophage apoptosis and the physiological role of miRNA-9.

## 2. Methods

### 2.1 Test process

**Figure 1.**
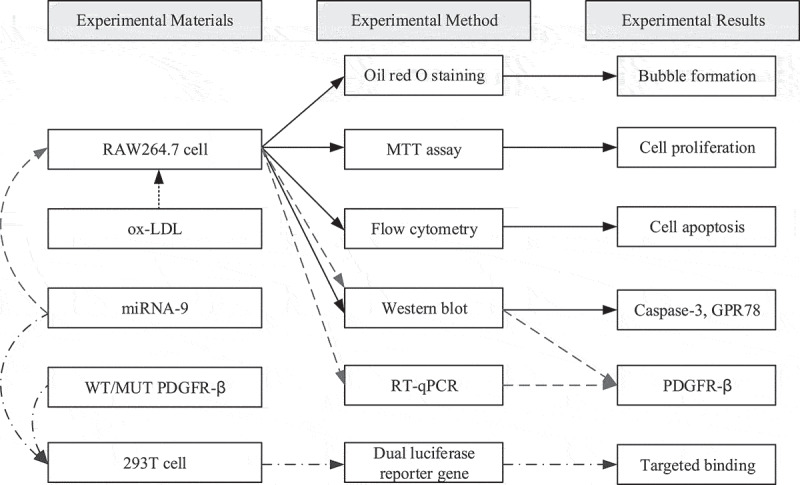
Diagram of the test process (arrows with different colors and different line types referred to different test processes)

The specific test process of this study is shown in Figure 1.

**Figure 2.**
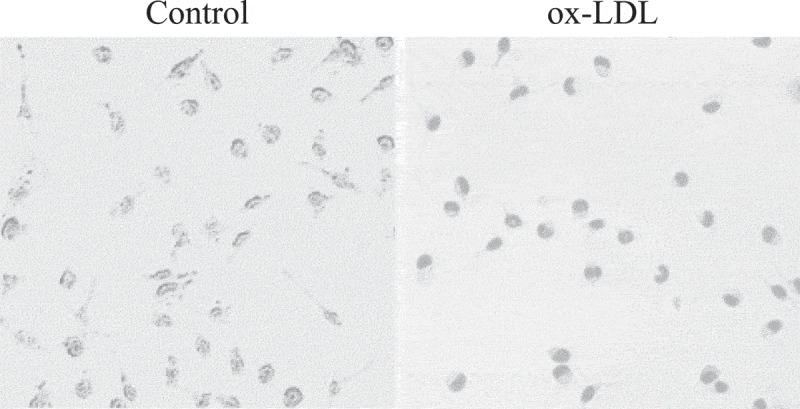
Observation of macrophage RAW264.7 oil red O staining (×100)

**Figure 3.**
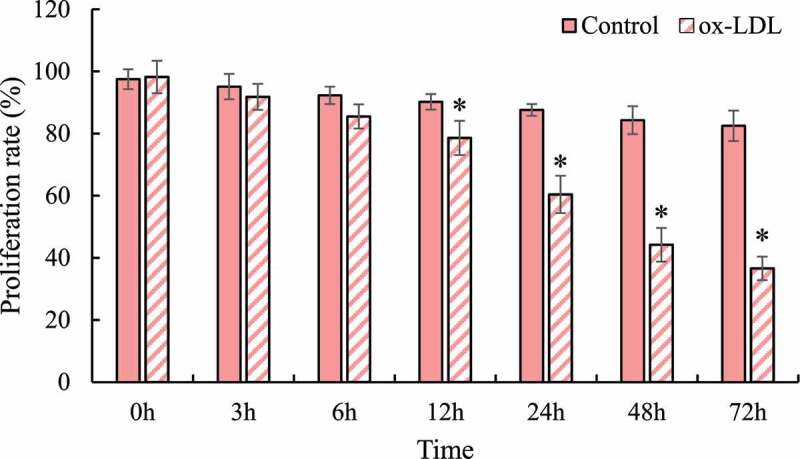
Macrophage RAW264.7 proliferation detection (* indicated that the difference between groups was statistically significant, *P* < 0.05)

### 2.2 Cell culture and grouping

RAW264.7 macrophages were cultured using Royal Park Memorial Institute (RPMI) 1640 medium (Gibco, US) containing 10% fetal bovine serum (FBS, Gibco, US) and 1% Distreptomycin (Gibco, US), cultured in an incubator containing 5% CO2 at 37°C. The passage was carried out every 2–3 days. The cells in the logarithmic proliferation phase were seeded in a 6-well plate, and 100 mg/L ox-LDL was added for 24 hours after continuing to culture for 24 hours to induce foaming of macrophages, which was recorded as the ox-LDL group. The normal cells were defined as controls (Control group).

### 2.3 Oil red O staining

After normal cells and ox-LDL induction, the supernatant was removed, the cells were washed 3 times with phosphate buffer, and then fixed with 10% formaldehyde reagent for 30 minutes. After washed the cells with phosphate buffer, the cells were immersed in 60% isopropanol solution for 5 minutes, and then added with Oil Red O for staining in a 60°C oven for 30 minutes. After washed with distilled water, the cells were added with hematoxylin for 5 minutes, washed with distilled water for bluing for 3 minutes, dried, taken photos, and observed under a fluorescence microscope.

### 2.4 3-(4,5-Dimethylthiazol-2-yl)-2,5-diphenyltetrazolium bromide (MTT) to detect cell proliferation

The cells were seeded in a 96-well plate, and three replicate wells were set in each group after group treatment. Then, after added with 10 μL of MTT reagent (Abcam, UK) with final concentration of 0.5 mg/mL, the cells were incubated for 4 hours in an incubator containing 5% CO2 at 37°C. After the cell supernatant was discarded, the cells were rinsed with phosphate buffer, and added with 150 μL of Dimethyl sulfoxide reagent to dissolve. Then, the mixture was shaken in a horizontal shaker at room temperature for 10 minutes, and then the optical density of the cells in each well at a wavelength of 490 nm was measured using a microplate reader.

### 2.5 Flow cytometry to detect cells apoptosis

According to Annexin V-Fluorescein Isothiocyanate (FITC)/Propidium iodide (PI) kit (Sigma, USA), cell apoptosis was detected [7]. After the cells were cultured for 48 hours, they were washed twice with pre-cooled phosphate buffer, and added with 500 μL of buffer to mix. Next, they were added with 10 μL of Annexin V-FITC reagent and 5 μL of PI reagent to mix, and incubated for 10 min in the dark. The flow cytometry was used to detect the rate of apoptosis. Each group of cells was set up with 3 multiple wells, and all experiments were repeated 3 times.

### 2.6 Real-time fluorescence quantification polymerase chain reaction (RT-qPCR)

The miRNA-9 mimics and inhibitors were transfected into macrophages RAW264.7. An appropriate amount of Trizol reagent was added to the treated cells to lyse the cells. After the chloroform reagent was added for 10 minutes, the solution was centrifuged at low temperature and high speed for 15 minutes to collect the supernatant. After adding an equal volume of isopropanol and mixing, the supernatant was allowed to stand at room temperature for 5 minutes, and then centrifuged at low temperature and high speed for 10 minutes to take the precipitate. The precipitate was washed with a pre-cooled 75% ethanol solution and dried naturally, added an appropriate amount of RNase-free ultrapure water to dissolve, and stored in a refrigerator at −80°C. Reverse transcription of complementary deoxyribonucleic acid (cDNA) was performed according to the instructions of the cDNA reverse transcription kit (Takara, Japan). The cDNA was undertaken as a template, and the target gene was quantified according to the instructions of the qPCR detection kit (Takara, Japan). With glyceraldehyde-3phosphate dehydrogenase (GAPDH) gene as an internal reference, the relative expression level of the target gene was calculated according to 2^−ΔΔCT^ [8].

### 2.7 Western blot

Each group of RAW264.7 cells after treatment was taken and added with Radio-Immunoprecipitation Assay lysis solution for cell lysis. According to the Bicinchoninic acid method (Beyotime Biotechnology, China) for quantitative detection of cell protein levels, the protein concentration was calculated by drawing a standard curve. The sample protein was collected, added with the same amount of dodecyl sulfate and sodium salt-Polyacrylamide gel electrophoresis (SDS-PAGE) protein loading buffer, and heated in a water bath at 100°C for 10 minutes to denature the protein. Then, 12% sodium dodecyl sulfate polyacrylamide gel electrophoresis (SDS-PAGE) concentrated gel was added for protein electrophoresis. On ice, the Polyvinylidene fluoride (PVDF) membrane was adopted for protein transfer. After washed with phosphate buffer solution tween (PBST), the membrane was immersed in a PBST solution containing 5% skimmed milk powder, and sealed at room temperature for 2 hours. Then, it was added with diluted caspase-3 (1:800), glucose regulated protein 78 (GRP78, 1:800), and PDGFR-β (1:800) primary antibody (Invitrogen, USA) to incubate overnight in a refrigerator at 4°C. After washed with PBST, the membrane was added with diluted horseradish peroxidase labeled secondary antibody (1:1000) to incubate for 2 hours at room temperature. Again after the membrane was washed with PBST, the chemiluminescent reagent was added to react for 3 minutes and develop in a gel imager. The Image J software was used to analyze the optical density value of the protein band, and the β-actin gene was used as a control to detect the relative expression level of the target protein.

### 2.8 Dual luciferase activity test

The day before transfection, 293 T cells were seeded in 96-well plates, and the cells were cultured in dulbecco’s modified eagle medium (DMEM) medium containing 10% FBS and 1% distreptomycin. Transfection was performed when the confluence was about 50%, and the transfection reagent Lipofectamine 2000 was diluted with serum-free cell culture medium. The miRNA-9 mimics and wild-type/mutant PDGFR-β expression plasmids were added to 293 T cells to mix at room temperature and let stand for 20 minutes. Subsequently, 50 μL of transfection complex was added to each well to culture in a cell incubator for 8 hours, and the Dual-Luciferase reporter assay system (Promega, USA) was used for dual-luciferase reporter gene analysis [9]. A multifunctional microplate reader was adopted to detect Renilla luciferase and firefly luciferase activity, and the ratio was calculated to obtain the relative luciferase activity.

### 2.9 Statistical Methods

The test data were all expressed as mean ± standard deviation, and the difference between groups was compared by one-way analysis of variance. Statistical analysis was done using the SPSS 19.0 software. *P* < 0.05 meant the difference was statistically significant.

## 3. Results

This study discussed the mechanism of ox-LDL-induced macrophage RAW264.7 and explored the targeted regulation of miRNA-9 on PDGFR-β, hoping to find potential molecular targets and provide experimental data for the treatment of atherosclerosis and other diseases.

## 3.1 Ox-LDL induced foaming of macrophages RAW264.7

Oil red O staining was used to observe the foaming changes of normal macrophages induced by RAW264.7 and ox-LDL (as shown in Figure 2). The number of stained lipid droplets in the control cells was small; while the cell volume increased after ox-LDL induction, the shape was more rounded, and a large number of lipid droplets could be clearly seen in the cytoplasm.

## 3.2 Ox-LDL inhibited the proliferation of macrophages RAW264.7

MTT was applied to detect the difference in the proliferation activity of normal macrophages RAW264.7 and ox-LDL at different times after induction (as shown in Figure 3). With the increase of culture time, the proliferation activity of each group of cells showed a gradual decline trend, and the decline trend of cell proliferation activity after ox-LDL induction was more obvious. The proliferation rate of macrophages RAW264.7 after 12 h, 24 h, 36 h, and 72 h induced by ox-LDL was significantly lower than that of the control group, and the differences were statistically significant (*P* < 0.05).

## 3.3 Ox-LDL induced apoptosis of macrophages RAW264.7

**Figure 4.**
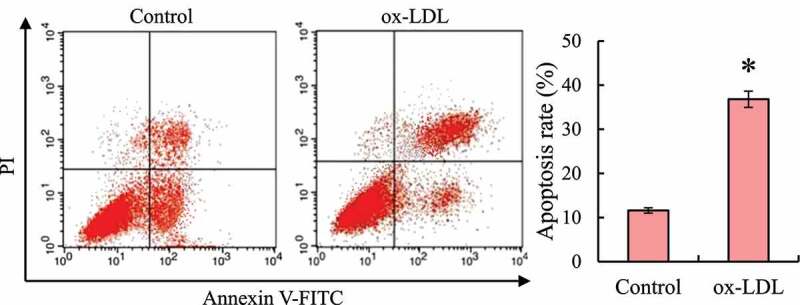
Macrophage RAW264.7 apoptosis detection (* indicated that the difference between groups was statistically significant, *P* < 0.05)

Flow cytometry was used to detect the apoptotic state of macrophages RAW264.7 induced by ox-LDL (as shown in Figure 4). Compared with the control group, the number of early cell apoptosis and necrosis in the ox-LDL group was significantly increased. After statistics, it was found that the total apoptosis rate of macrophages RAW264.7 after ox-LDL induction was significantly higher than that of the control group, and the difference was statistically significant (*P* < 0.05).

### 3.4 Effect of ox-LDL-induced macrophage RAW264.7 on the expression of caspase-3 and GRP78 protein

**Figure 5.**
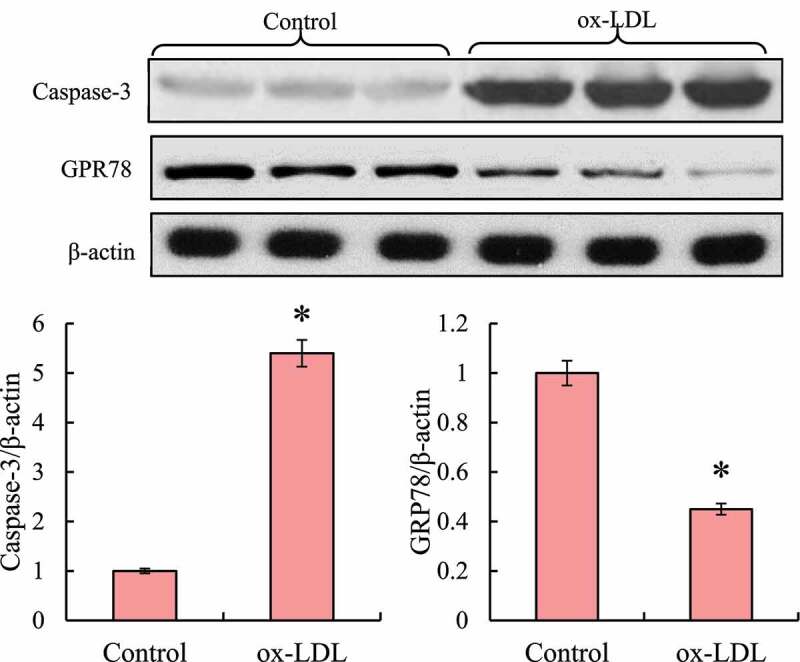
Detection of Caspase-3 and GRP78 protein expression levels in macrophages RAW264.7 (* indicated that the difference between groups was statistically significant, *P* < 0.05)

Western blot was used to detect the changes in the expression levels of caspase-3 and GRP7 proteins in macrophages RAW264.7 induced by ox-LDL (as shown in Figure 5). After ox-LDL induction, the expression of Caspase-3 in macrophages RAW264.7 was up-regulated, while the expression of GRP78 was down-regulated. After the relative expression level was calculated, it was found that compared with the control group, the Caspase-3 protein expression level in macrophages RAW264.7 was significantly reduced after ox-LDL induction, while the GRP78 protein expression level was significantly increased, showing statistically significant differences (*P* < 0.05).

### 3.5 The effect of miRNA-9 on the expression level of PDGFR-β

**Figure 6.**
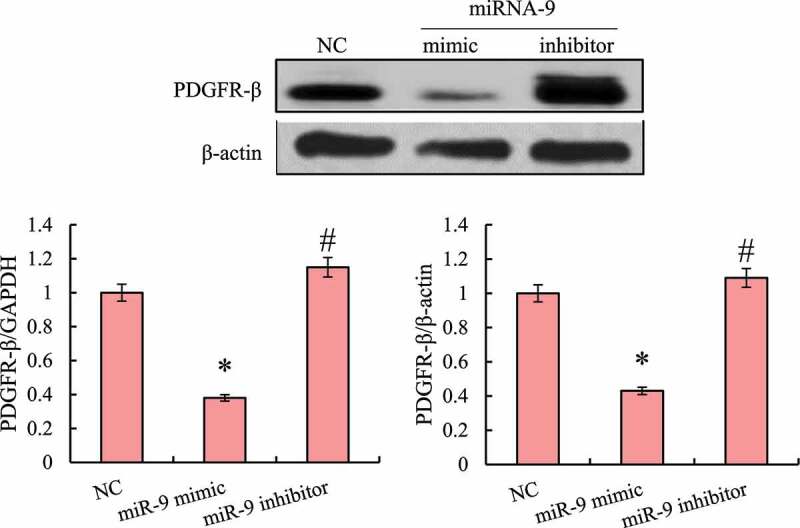
Detection of PDGFR-β mRNA and protein expression levels in macrophages RAW264.7 (* indicated that the difference between groups was statistically significant, *P* < 0.05)

RT-qPCR and Western blot were used to detect the effect of transfection of miRNA-9 mimics and inhibitors on the expression levels of PDGFR-β mRNA and protein in macrophages RAW264.7 (as shown in Figure 6). A blank transfection reagent was undertaken as a negative control (NC), it was found that after transfection of miRNA-9 mimics, the expression of PDGFR-β mRNA and protein in the cells was significantly down-regulated, and it can increase the expression levels of PDGFR-β mRNA and protein in cells after transfection with miRNA-9 inhibitors, and the difference was statistically significant (*P* < 0.05).

### 3.6 Target prediction of miRNA-9 regulating PDGFR-β

**Figure 7.**
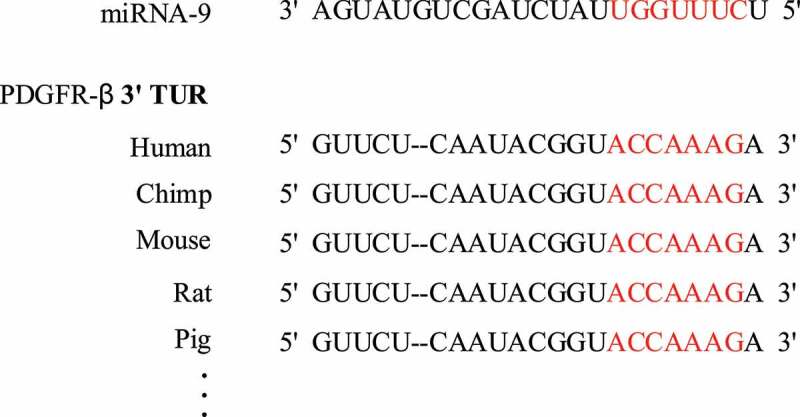
Target prediction results of miRNA-9 and multi-species PDGFR-β 3ʹ UTR region (red bases were the target binding sites)

The TargetScan, miRbase, miRDB, and other data were adopted to predict the target-binding sites of miRNA-9 and PDGFR-β (as shown in Figure 7). It was found that miRNA-9 had a 7-base complementary pairing with the 3ʹUTR region of PDGFR-β, and the targeted binding site was conserved in many species including humans, chimpanzees, mice, rats, and pigs.

### 3.7 Detection on miRNA-9 targeted regulation of PDGFR-β using dual luciferase reporter gene

**Figure 8.**
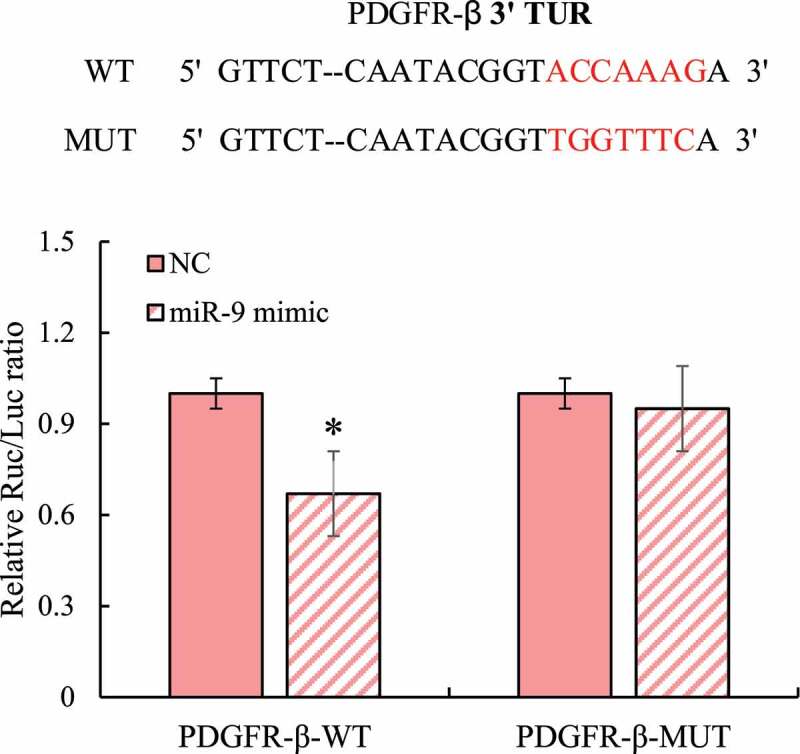
Results of dual luciferase reporter gene detection (red bases were wild-type and mutant-type binding sites; and * indicated that the difference between groups was statistically significant, *P* < 0.05)

A mutant plasmid of the binding site of the PDGFR-β 3ʹUTR region was constructed, and the wild-type/mutant PDGFR-β expression plasmids were co-transfected with miRNA-9 mimics into 293 T cells. The relative activity of the cellular dual luciferase was detected (as shown in Figure 8). After the wild-type PDGFR-β and mutant PDGFR-β were co-transfected with the negative control, the dual-luciferase activity did not change, while the luciferase activity of the mutant PDGFR-β and miRNA-9 mimic co-transfected did not change obviously. After wild-type PDGFR-β and miRNA-9 mimics were co-transfected, the ratio of dual-luciferase activity in the cells was significantly reduced, and the difference was statistically significant (*P* < 0.05).

## 4. Discussion

Ox-LDL is a key factor in the occurrence and development of AS [10]. Studies have confirmed that after ox-LDL induces foaming of macrophages and smooth muscle cells, it can play a role in regulating cell proliferation and apoptosis [3]. Monocyte-derived macrophages engulf many ox-LDL to form foam cells to promote lipid deposition, and oxidative damage of cell membrane to promote cell apoptosis. Apoptosis cells causing secondary inflammatory response and necrosis promote plaque instability by releasing inflammatory factors such as matrix metalloproteinases tumor necrosis factor TNFα and interleukin 1β [11]. Studies showed that ox-LDL can induce macrophages apoptosis and inhibit the release of inflammatory factor TNF-α, IL-6, and inflammatory mediators NO [12]. The results of this study showed that with the prolongation of ox-LDL induction time, the proliferation activity of macrophages RAW264.7 was significantly reduced, and the apoptosis rate of cells was increased. This may be because ox-LDL induction can cause damage to macrophages, and the damage increases with time. Ox-LDL can cause macrophage injury, and the degree of injury increased with the increase of ox-LDL concentration. Recent studies showed that ox-LDL can induce macrophages to produce erythrocyte sedimentation rate (ESR) in a dose-dependent manner, and induce apoptosis through caspase-12 and ESR-C/EBP homologous protein (CHOP) stimulus of caspase-9 and caspase-3 [13,14]. The results of this study showed that ox-LDL induction could increase the expression level of Caspase-3 protein in macrophages and reduce the expression of GRP78.

With the deepening of miRNA research, these ubiquitous small molecules play a wide role in the regulation of eukaryotic gene expression. Hundreds of miRNAs were found in species of nematodes, flies, mice, and human, most of which have the same characteristics as other molecules involved in regulating gene expression. The expression levels of miRNAs in different tissues and different stages of development are significantly different. The expression patterns of miRNAs have phase and timing of differentiation [15], suggesting that miRNA may exist as molecules involved in regulating gene expression. MiRNA plays a role through two main mechanisms: the first is completely or nearly completely paired with the target RNA, playing a similar role as interfering RNA, inhibiting its translation, and degrading the target mRNA [16]. The second inhibits the start-up and extension stages of target mRNA translation through incomplete complementary binding to the 3UTRs region of mRNA, resulting in gene silencing [17]. The abnormal expression of miRNA-9 is related to the promoter, which has an important influence on gene expression. Promoter methylation or demethylation is an important way to regulate gene expression [18].

As a core node and new node for understanding the complex and recessive pathogenesis of diseases, miRNA is attracting more and more attention [19]. PDGFR is a type of tyrosine protein kinase receptor on the cell membrane, which can be activated by growth factors, such as PDGF and TGF-β to promote cell growth, transformation, and migration [20]. Studies have confirmed that the PDGFR-β signaling pathway plays an important role in promoting the proliferation of cardiac fibroblasts and the synthesis of collagen [21], and miRNA-9 can inhibit the proliferation of cardiac fibroblasts [22]. In order to verify the interaction between miRNA-9 and PDGFR-β, this study overexpressed miRNA-9 and found that the expression of PDGFR-β mRNA and protein in macrophages was significantly inhibited. Therefore, it was speculated that there was a targeted regulation effect between miRNA-9 and PDGFR-β. In order to verify this hypothesis, this article used online software to predict the targeting effect of miRNA-9 and PDGFR-β, and found that miRNA-9 can target and bind to the 3ʹUTR region of PDGFR-β and the sequence was conservative across multiple species. Subsequently, the dual-luciferase reporter gene system was used to verify that miRNA-9 can target PDGFR-β.

## 5. Conclusion

The RAW264.7 macrophage-derived foam cell model was constructed in this study to explore the mechanism of ox-LDL-inducing macrophage apoptosis and the mechanism of miRNA-9 inhibiting the expression of target gene PDGFR-β. The effect of ox-LDL induction of the expression of lipid metabolism-related receptor mRNA in the apoptosis of macrophage-derived foam cells was studied by real-time quantitative PCR. In addition, the expression of PDGFR-β and protein was detected by RT-qPCR, Western blotting, and immunohistochemistry. ox-LDL could induce the foaming of macrophages RAW264.7, inhibit cell proliferation, and promote apoptosis. miRNA-9 can target and bind to the 3ʹUTR region of PDGFR-β, thereby inhibiting the expression of PDGFR-β.

## Research limitations

In this study, ox-LDL induction and miRNA-9 targeted regulation of PDGFR-β were verified only at the cellular level, and no animal experiments were performed. In addition, it failed to verify whether miRNA-9 can improve or aggravate atherosclerosis through targeted regulation of PDGFR-β.

## Future work

It would be devoted to exploring the mechanism of miRNA-9 expression on ox-LDL-induced foaming and apoptosis of macrophages, and exploring the effect of knocking out PDGFR-β gene on animal models of atherosclerosis.
